# A Cross-Sectional Study of Horse-Related Injuries in Veterinary and Animal Science Students at an Australian University

**DOI:** 10.3390/ani5040392

**Published:** 2015-09-25

**Authors:** Christopher B. Riley, Jessica R. Liddiard, Kirrilly Thompson

**Affiliations:** 1 Institute of Veterinary, Animal and Biomedical Sciences, Massey University, Tennent Drive, Palmerston North, 4442, New Zealand; 2 School of Animal and Veterinary Sciences, University of Adelaide, Roseworthy, SA 5371, Australia; E-Mail: jessicaliddiard23@gmail.com; 3 Appleton Institute, Central Queensland University, Adelaide, SA 5034, Australia; E-Mail: kirrilly.thompson@cqu.edu.au

**Keywords:** horse-related, injury, accident, student, education, veterinary

## Abstract

Specific estimates of the risk of horse-related injury (HRI) to university students enrolled in veterinary and animal sciences have not been reported. This study aimed to determine the risk of student HRI during their university education, the nature and management of such injuries. A retrospective questionnaire solicited demographic information, data on students’ equine experience prior to and during their educational programs, and on HRI during their program of study. Of 260 respondents, 22 (8.5%) reported HRI (27 incidents). Including concurrent injuries the most commonly injured body parts were the foot or ankle (nine of 32 injures), the upper leg or knee (eight of 32), and hands (three of 32). Trampling and being kicked by a hind limb were each associated with 30.4% of HRI, and 13% with being bitten. Bruising (91.3% of respondents) and an open wound (17.4%) were most commonly described. No treatment occurred for 60.9% of incidents; professional medical treatment was not sought for the remainder. Most incidents (56.5%) occurred during program-related work experience placements. Although injury rates and severity were modest, a proactive approach to injury prevention and reporting is recommended for students required to handle horses as part of their education. Student accident and injury data should be monitored to ensure effective evaluation of risk-reduction initiatives. The risk and nature of university student horse-related injury (HRI) was studied. Of 260 students, 22 (8.5%) reported HRI (27 incidents). Including multiple injuries, reports described involvement of the foot or ankle (nine of 32 injures), upper leg or knee (eight of 32), and hands (three of 32). Trampling (30.4%) and being kicked (30.4%) accounted for most HRI. The injuries were usually bruising (91.3%) or an open wound (17.4%). Most (60.9%) injuries were untreated; professional medical treatment was not sought for the rest. Most incidents (56.5%) occurred during program-related off-campus work experiences. A proactive approach to injury prevention is recommended for students handling horses.

## 1. Introduction

Horses are powerful and frequently unpredictable animals, capable of moving at high speeds and of generating great force with a single kick [1,2]. Due to the combination of behavioral characteristics, nature of responses to adverse stimuli, speed, power, and size of these animals, people that interact with horses professionally or recreationally are at risk of severe or fatal injuries. Studies of equine-related injury have been conducted for people engaged in a diverse range of equestrian activities, but the majority of these have investigated the risk of injury to riders [3,4]. In comparison, research on occupational injury by horses within the veterinary profession and other equine industries is limited [2,5,6,7]. A cross sectional survey of Australian veterinarians (the Health Risks of Australian Veterinarians study; HRAV) found that most large animal (65% equine and/or food animal), and mixed animal (59%; large and companion animals) veterinarians had suffered chronic musculoskeletal or severe acute injuries, placing them at the highest risk of significant injuries [2,5,6]. Significant injuries were classified as an incident resulting in hospital admission, or having a substantial detrimental effect on the ability to work. Of the serious injuries reported, 29% were directly equine associated [2], of which 70% occurred despite safety precautions that were claimed to have been in place, indicating that the latter may have been insufficient or incorrectly applied [5]. Such findings are not unique to Australian veterinarians, and studies in other countries have found similar concerning statistics [8,9].

Currently protocols are in place in many universities with equine educational programs to ensure the safety of students and staff. These include testing the suitability of horses for teaching, hazard evaluation, and incident reporting systems, though few protocols have been published [10,11]. More information on risk factors and prevalence is needed to develop specific and effective evidence-based recommendations for minimizing horse-related injuries (HRI) [10,12]. Reports of occupational HRI for experienced veterinarians provide useful information, but may not be generally applicable to university students enrolled in programs requiring exposure to horses, as the latter have more variable levels of equine experience and interest. At the University of Adelaide, students enrolled in the animal (AnSci) and veterinary science (VS) programs may elect (AnSci and VS) or are required (VS) to obtain practical experience with horses as part of their educational programs. The authors hypothesized that injury rates for students at the University of Adelaide differed from those that of reports for practicing veterinarians and others involved in the equine industry from similar cultural backgrounds. The objectives of this study were to determine the prevalence of injury for university students exposed to horses during structured and unstructured learning activities associated with their program of education, and to identify particular risk factors. The authors found low rates of injury, but a lack of action taken to see medical evaluation in response to these injuries raises concerns about risk culture in Australian students, and its possible carry over into professional life [13].

## 2. Experimental Section 

The study followed the recommended assessment procedures for studies with low ethical risk approved by the Human Research Ethics Committee of the University of Adelaide.

### 2.1. Background Information Collection

In order to clarify the background conditions underpinning the experience of the survey participants, current safety protocols for on campus and extramural (equine work experience) learning activities involving horses were reviewed. The campus Health and Safety Officer (HSO) was also asked to provide a summary of the number of HRI formally reported by students during the five years preceding the survey

### 2.2. Survey Data Collection

Students were approached and directly invited to participate in the paper-based survey within the daily class schedule. This occurred over a 29-day sampling period. A response rate of 60% from each year cohort of students within each educational program was anticipated. At the conclusion of the initial survey participation request period, a final attempt was made to acquire results from non-responding students by emailing the survey to each of the cohorts with a description of the study to be undertaken. No identifying information was recorded on the survey or by other means. 

Students enrolled in the undergraduate Animal Science (AnSci), Veterinary Bioscience (VetBio), or Masters by coursework Doctor of Veterinary Medicine (DVM) programs at the University of Adelaide (South Australia) for the 2012 academic year were considered eligible for the study (n = 461). An open source sample calculator was used to determine an appropriate target for the number of respondents from this study population to obtain a survey error (type I) no higher than a 5% [14]. It was calculated that to be within a 95% confidence interval 214 respondents were required, and to be within a 99% confidence interval 279 respondents were required [14].

An anonymous retrospective questionnaire was created consisting of 33 questions in three sections: (1) background demographic information, (2) qualitative and quantitative data regarding attitudes and experiences of students prior to and during their program of education, and (3) incidence of horse-related injury, reporting and management. Background demographic information collected included gender, age, educational program and year level, hours of horse exposure in practical equine courses completed on-campus, and length of time spent at equine work experience placements outside of the university campus. Student attitudes and experiences were solicited and semi-quantitative data was obtained using rating scale questions in which the scale range was from 0 to 100. These questions surveyed the students’ interest and prior contact with horses, and perceptions of safety procedures.

Finally, the incident report section of the survey was used to identify injury incidence, type, mechanism, severity, and the various contributing factors as perceived by the students. To ensure consistent interpretation of quantitative responses, where a range value was provided the mean of the range was utilized for the purpose of analysis. For example if a respondent commented that they had 10–20 hours of contact with horses per month the mean value of 15 was utilized. Scaled questions offered a semi quantitative range of responses from 0 (most negative or greatest magnitude of disagreement) to 100 (most positive or greatest magnitude of agreement), and results were interpreted at a minimum interval of five units. Where only a year value was given in response to how much time the respondent had spent with horses prior to the program, the respondent was conservatively estimated to have had two hours of contact with horses for each day of the time indicated to generate a final approximation for analysis. Alternatively, where insufficient information was provided to make such an assumption (e.g., “a lot”), the response was neither transcribed nor included in the data analysis.

### 2.3. Data Analysis

Associations between categorical variables including program type, program year, gender and the occurrence of injury were evaluated by the Chi-squared test (considered significant at *p* < 0.05); where the expected values were <5, Fishers exact test was performed. The distribution of values for continuous variables (age in months, duration of equine experience before enrollment, and equine contact time before and during enrollment) was evaluated by the Shapiro-Wilk test; none were normally distributed. The effects of these continuous variables on the occurrence of injury were analyzed using the Kruskal-Wallis test; differences were considered significant at *p* < 0.05.

## 3. Results

### 3.1. Safety Protocols-On Campus Safety and Teaching Horse Assessment

The review of safety protocols in use at the time of the survey found that horses acquired for live animal teaching on the university campus were identified by the Teaching Services Technical Manager, and examined by a registered staff equine veterinarian for health, temperament, and ease of handling. Suitable horses were then transported to the campus for two to four weeks of handling and further evaluation of temperament by equine teaching staff. Horses that failed to adapt to the teaching environment, or showed evidence of recurrent intemperate behavior during teaching activities, were removed from the teaching herd. Qualified staff supervised all equine-related teaching activities on-campus. Formal qualifications held by the staff ranged from Australian equine industry certification in equine handling and training, to graduation from an AnSci or veterinary degree program. The staff to student ratio for classes with horses was ~1:6. All sessions involving horses required students to read and understand standard operating procedures for basic handling and restraint, and the veterinary technical procedures developed for the learning activities. Equine related teaching was delivered in purpose-built yards, stalls, and crushes. All adverse incidents observed by staff or reported by students were noted and forwarded to the campus HSO. 

### 3.2. Safety Protocols-Extramural Equine Safety

A designated staff member was assigned to coordinate student placement (work experience) agreements with host-providers and manage extramural placements from the campus. Placement hosts were required to provide relevant experiences (animal husbandry for AnSci and VetBio students; veterinary clinical training for DVM students), to provide a local safety induction to students, and to adhere to Australian legal requirements for work place health and safety. Feedback on extramural placements was sought from hosts and students, and responded to directly by the coordinator.

### 3.3. Demographic and Background Data

Of 481 eligible AnSci and VS students, 260 (54.1%) returned surveys (Table 1). The resulting calculated sampling error was 4.1% at the 95% confidence level [14]. Female students constituted 219 of 264 (83%) of respondents (compared to 81% in the eligible AnSci and VS student population), and males constituted 45 of 264 (17%) respondents (compared to 19% in the eligible population). The median age of the participants was 21.5 years (range 17.8 to 42.5). For background questions not related to describing an incident, response rates per question ranged from 78 to 100%. Questions pertaining to an incident (22 students; 23 of 27 incidents described) had a 100% response rate except for the date of the incident (74% response rate).

**Table 1:** The number of respondents, demographic data, survey response rate for each year level and program.

	Animal Science			Veterinary Bioscience			Doctor of Veterinary Medicine		Total
Year of Program	1	2	3	1	2	3	1	2	
2012 Enrolments	119	48	54	42	65	64	53	36	481
Number Surveyed	33	26	23	29	32	45	46	26	260
% of Class Surveyed	27.7	54.2	42.6	61.9	49.2	70.3	86.8	72.2	54.1

**Figure 1:**
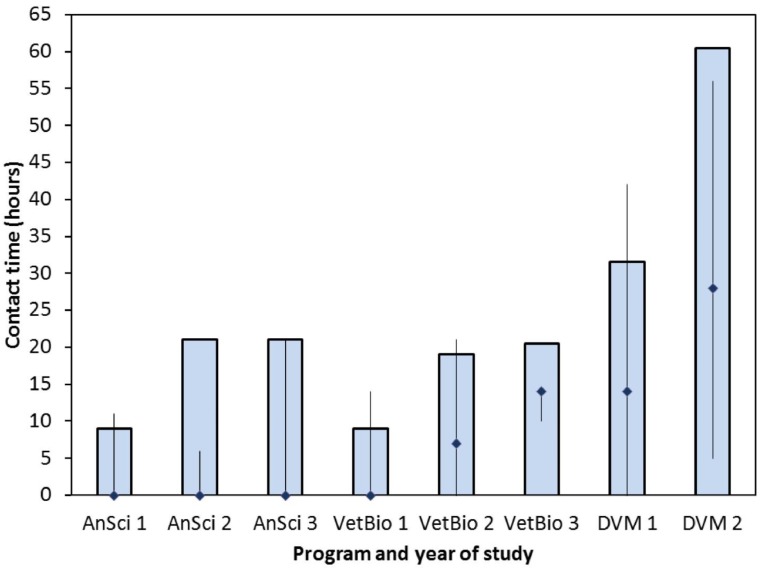
Contact time spent with horses during on-campus instruction (blue column) and extramural equine work experience placements (diamond = median; vertical bars indicate range) for Animal Science (AnSci), Veterinary Bioscience (VetBio) and Doctor of Veterinary Medicine (DVM) students at the University of Adelaide. Note, on-campus contact time was prescribed by the courses in which students were enrolled.

Structured university courses with an equine practical component were compulsory and the duration of on-campus equine contact was constant within each cohort (Figure 1). On-campus class contact time with horses of AnSci and VetBio cohorts were approximately equivalent, whilst DVM students had a significantly greater amount of on-campus class equine contact time in comparison (*p* < 0.05; Figure 1). The time spent on extramural placements (a compulsory requirement of the VetBio after the first year and the DVM programs) varied among veterinary students (Figure 1). A minimum two-week extramural placement to gain experience in equine handling and husbandry was required of the VetBio program respondents, and a minimum five-week extramural placement for equine clinical experience was required of the DVM program respondents. Comparatively, there was no compulsory requirement for an equine based extramural placement in the AnSci program; approved placements were elective.

Sixty percent (156 of 260) of respondents reported contact with horses prior to beginning their university program. The approximate duration of this experience varied widely (mean 340 ± 811 days). Only 41 (16%) of students indicated that working with horses strongly influenced their decision to enroll in their current program (indicated by a score >70/100). These values did not differ significantly between AnSci and VS students, or among year levels within programs. A significant difference between injured and non-injured students in the duration of equine experience before enrollment was not identified.

### 3.4. Injury Data

Of the 260 respondents, 8.5% (22) reported a total of 27 incidents resulting in injury (Table 2). Only details of 23 incidents (one per student in 21 cases, and one reported the details of the two most recent incidents) were provided for analysis. Only one incident was reported to the campus HSO during 2010 to 2012; HSO records were not available for the three years prior to 2010. Most injured students were female (90.9%; 20/22), and 9.1% (2/22) were male (Table 2). There was no statistically significant gender association with the risk of injury. Students that had experienced a horse-related injury were found to have a median age of 22 years, which was not significantly different than non-injured respondents (21.5 years). 

There was a significant association between student contact time (non-linear) with horses during programmed educational activities, and an increased risk of injury (*p* = 0.016) that could not be differentiated from associations with the program of study.

**Table 2:** The injury response rates and relative risk for each year level and program.

	Animal Science			Veterinary Bioscience			Doctor of Veterinary Medicine		Total
Year of program	1	2	3	1	2	3	1	2	
Number of injured students	2	0	0	0	2	7	5	6	22
Number of incidents	2	0	0	0	2	7	5	7	23
Relative risk by year	0.06	0	0	0.07	0.16	0.02	0.11	0.04	
Relative risk by program		0.02			0.14 ^1^			0.12 ^2^	

^1^ The year in which the injury occurred was not reported in one case; ^2^ The year in which the injuries occurred were not reported in four cases.

Of the 23 incidents described, AnSci students reported two (8.7%), the VetBio students reported nine (39.1%), and the DVM students reported 12 (52.2%) (Table 2). The AnSci students were less likely to sustain HRI than VetBio and DVM (*p* = 0.013), and the two AnSci incidents described involved riding and colliding with a gate during their extramural placement on farms. The DVM students were associated with a higher risk of HRI than the AnSci students (*p* = 0.038); the difference between VetBio and DVM students was not statistically significant. In only 17 incidents did injured students report the year in which the incident occurred, and the relative risk was highest in the second year of the VetBio and first year of the DVM programs (Table 2).

The anatomic locations and nature of reported injuries sustained are summarized in Table 3. Six respondents reported more than one type of tissue injury due to their HRI. The most common mechanisms of injury were a horse trampling the foot or ankle, being kicked by a hind limb, or being bitten. The most commonly reported types of injuries were bruises or an open wound; three students reported concurrent bruising and a laceration. 

Most injured students, (60.9%; 14) did not treat the injury and remaining injuries (39.1%; nine) were self-treated. There were no incidents for which first aid treatment was sought, a doctor was seen, or a student admitted to hospital. None of the injured respondents reported requiring time off from university study or work following injury and only one student (4.3%) with a muscular lower back injury of unreported duration, indicated that she had not fully recovered from the incident. A median of 14 days (range 0 to 60 days) was reported for the remaining students to fully recover from their injuries.

**Table 3:** Description of the injuries sustained by students including bodily location, mechanism of injury, and nature of injury.

Factor		Number of Injuries	Percentage of Incidents
Affected Bodily Location	Foot/Ankle	9	39.1
	Upper Leg/Knee	8	34.8
	Hand	4	13.0
	Lower Leg (Calf/Dorsal Tibia)	2	4.3
	Head	1	4.3
	Face	1	4.3
	Forearm	1	4.3
	Lower Back	1	4.3
	Other	1	4.3
	Eye	0	0
	Upper Arm/Shoulder	0	0
	Neck	0	0
	Spine	0	0
	Pelvis	0	0
	Total injuries reported	28	-
Mechanism of Injury	Trampled	8	30.4
	Kicked by hind limb	7	30.4
	Bitten	3	13.0
	Fall while riding	2	8.7
	Struck by forelimb & Crushed by body	1	4.3
	Knocked over	1	4.3
	Struck by horse’s head	1	4.3
	Total mechanisms reported	23	-
Nature of Injury	Bruising/Soft Tissue Injury	20	91.3
	Open Wound	4	17.4
	Muscle or Tendon Injury	2	8.7
	Rope Burn	2	8.7
	Non-Specified Internal Injury	1	4.3
	Crushed Tissue Injury	1	4.3
	Muscular Strain	1	4.3
	Fracture	0	0
	Intracranial injury	0	0
	Total injuries reported	31	-

Incidents occurred most frequently during extramural placements to obtain experience in equine husbandry (56.5%; 13 of 23 incidents). All injuries to second-year VetBio students and first-year AnSci students occurred during an extramural placement (Table 2), whereas for the DVM and other VetBio program years injuries occurred in approximately equivalent numbers on and off campus. No injuries associated with extramural placements at veterinary practice facilities were reported to the HSO. Nine adverse horse-related incidents (39.1%; nine of 23 incidents) occurred on the premises of the university during teaching activities, and one (4.3%) at an equine event unrelated to an educational program. Specifically, nine (39.1%) HSI occurred in handling yards or a fenced enclosure, eight (34.8%) in a stable or barn, two (8.7%) in an open paddock, two in an arena (8.7%), one (4.3%) in a horse float (trailer), and one in a non-specified location.

The activities or actions being undertaken by the students at the time of injury included standing near a horse (26.1%; six incidents), performing a handling or husbandry procedure (21.7%; five cases; e.g., moving horses or rounding up livestock on horseback), or performing a non-invasive physical examination procedure (17.4%; four incidents; e.g., auscultation of the chest). Walking near a horse, working with the horses’ limbs or feet, and catching a horse in a paddock were each associated with two incidents. Leading a horse in an outside location and trailering (floating) horses were each associated with a single incident. The activities for three incidents were not described. One or more factors were believed by respondents to be associated with their injury including resistance by the horse to handling (12 students; 52.2%), inexperience on their part (nine students; 39.1%), and seven admitted inattention on their part (30.4%). Seven (30.4%) believed that the incident occurred because the horse was in distress or fearful. Poor restraint and inattention by the handler were considered factors in four (17.4%) incidents, and the horse evading capture or poor staffing levels were each believed to contribute to a single incident each. For 12 (52.2%) incidents, respondents indicated that more than one factor might have contributed to the incident (range 0 to 4 factors).

In four (17.4%) incidents, it was the student’s perception that no safety precautions other than haltering or tying were being implemented at the time of the event. In the remaining 19 (82.6%) incidents, 15 (65.2%) of the students used protective footwear, three (13.0%) reported the horse was being restrained by an experienced staff member, two (8.7%) reported the horse was being restrained by an inexperienced peer. Additionally, two students used a nose twitch, two used another form of safety precaution outside of the defined categories, one (4.3%) stated the horse was sedated, and another used a helmet.

## 4. Discussion

Regardless of their experience levels, backgrounds and attitudes towards horses, it is compulsory for all American Veterinary Medical Association accredited university veterinary program students, and many animal science program students, to work with horses for some length of time within their educational programs. In comparison, qualified veterinarians exposed to horses have generally elected to follow this career path. The findings of the current survey support our hypothesis that when compared to reports for practicing veterinarians and others involved in the equine industry from similar cultural backgrounds, injury rates differ [2,4,5,6,7,15]. In contrast to equine industry reports, there were no severe injuries among students [3,4,15]. This result, to some degree, reflects favorably on the current procedures, facilities, environment, and staff for on-campus learning, and the selection of host locations for extramural experiences. However, HRI was reported, and the relatively lower rate should not be cause for complacency. An opportunity for comparison of these data with those of other comparable study populations is limited. However, given that attitudes and training with respect to animal safety might reasonably be expected to influence future professional practice, the relationship between the risk of HRI sustained by VS, and that of Australian qualified veterinarians warrants future study [13]. The overall prevalence of all HRI in university students was lower than the prevalence of 16.2% in 2800 qualified Australian veterinarians, but this might be expected as the exposure risk (time) for practicing veterinarians is higher [2]. The real difference in injury rates between the two groups may be greater if the HRAV had accounted for all degrees of injury, not just those that were classified as severe [2]. The design of the questionnaire used for the current study encouraged disclosure of all incidents irrespective of the severity of injuries sustained. The authors contend that this approach may be a more useful one than studies limited to only self-reported severe injuries or hospital admissions, to more accurately reflect the risk of HRI in other settings. 

Anecdotally underreporting of incidents is prevalent within the Australian veterinary profession and other equine industries, resulting in underestimation of rates of injury [7]. In agreement with other industry sector reports, formal incident reporting (to the HSO) by the current study population was lacking despite clear policies requiring the documentation of such events [4,7,13]. Injured students also failed to seek independent third party medical assessment. Such behaviors are of concern and may reflect Australian cultural and professional norms related to work practice, lack of safety awareness, and poor self-care [13,15]. It is recommended that greater emphasis be made within educational institutions to develop and monitor a non-punitive and supportive culture of accurate incident and hazard reporting among students and staff [16]. Based upon the data in this report, it is suggested that this process should extend to those incidents that occur during off campus extramural placements. To this end the risk assessment and injury reporting documentation should be provided to hosts and students required or electing to engage in these activities. This would enable a more accurate assessment of the risk of HRI so that evidence based hazard mitigation measures may be identified and taken.

In the current and HRAV study populations the rate of injury in males to females is proportionate to the distribution of gender [2]. Accordingly, a greater net number of females had sustained horse-related injuries in the current student population. In comparison a greater number of males sustain HRI in the qualified veterinary population due to greater proportion of males in equine and mixed practice at the time of the HRAV study [2]. This compares to a study of horse-related injury in Australian riders in which young female riders and older males were found to have the greatest risk of injury [15]. This is likely to be due to the demographics of amateur female riders, and more aged professional equestrians [15,16]. 

The significant relationship between contact time with horses within the university and the risk of HRI seems intuitive. However, given the limited data available for multivariate analysis, and the relatively uniform amount of equine contact time spent within each cohort, it was not possible to differentiate exposure time from the program-associated risk. Injuries occurred despite the university’s current measures to mitigate the risk of HRI. Nevertheless, most incidents occurred during extramural learning activities, and similar safety precautions are less likely to be implemented uniformly. Focused feedback following external placements that addresses these issues is required to evaluate this hypothesis. In lieu of such data, a review of each of the approaches to extramural risk mitigation is necessary to reduce the off campus exposure to HRI [17,18].

Student injuries were predominately sustained as a consequence of being trampled or being kicked by the hind limb, and most commonly resulted in a bruise or open wound. The bodily locations that were most commonly affected were the lower or upper limbs. In comparison, a larger proportion of serious injuries in Australian veterinarians are a result of kicks or strikes (79%), and a smaller proportion from being crushed or trampled (12.3%) [2]. They typically involve the lower extremities (33%); head and neck (26%), and upper extremities (20%), with fractures (27.8%) and bruising (27.6%) described as most common [2]. The findings of the current study are in agreement with a small study of unmounted rider HSI, with half of the riders also sustaining a contusion to the limbs [16]. In contrast facial injuries have also been commonly documented in the riders, and the reasons for this difference are unclear [19,20]. 

Over half of the HSI occurred at off campus locations for training required by their educational program, including all incidents for two cohorts that occurred during these extramural activities. Insufficient data (incidents) were available to statistically conclude that external placement constitutes an inherently high-risk situation for students. The comparative assessment of risk is confounded by the fact that the nature of the interactions and type of activities with horses on external placements differ from the exposure on-campus premises, as do facilities, and policies with respect to the incident review framework used to formulate the survey. For example, students most commonly reported being situated in a handling yard, stable, or barn at the time of injury. In comparison, qualified veterinarians were most likely to have sustained horse-related injuries in handling yards (37.7%) and stables (15.7%), but additionally in open paddocks (36.6%) [2]. It is possible that such differences arise from the limited or poorly maintained handling yards/crushes on Australian properties often faced by qualified veterinarians, compared to the recently purpose-built facilities at the university campus [7]. The most common safety precautions used by Australian veterinarians are physical restraint of the horse (34%) or hand-held reliance only (9%) [5]. Many large animal veterinarians are required to attend ambulatory visits alone and may not have an adequately skilled person to assist them [7]. In contrast, all on-campus equine-related teaching is supported by qualified staff [5,7]. However, students on external placements may face similar risk conditions as those experienced by practicing veterinarians; these conditions require further study. The authors agree with Jeyaretnam’s and others’ conclusions that a greater emphasis must be placed on determining the true risk of injuries and thus generating effective strategies to mitigate the hazards [5,21,22].

Currently Adelaide University students are required to wear protective boots during equine activities. However, the data indicated that ~35% of students are not compliant with this policy. Therefore further emphasis on ensuring that utilize sufficient protection against crushing injuries, and compliance with university clothing safety policy is warranted both on and off campus. Consideration should be given to the design and use of coveralls with protective padding over the thighs and knees such as that used for other competitive and recreational pursuits. Stock handling gloves should be worn for horse handling to prevent rope burn injuries. Whilst currently available safety equipment such as chest protection and helmets have been suggested to decrease the likelihood of injury [5] the effectiveness of this equipment for university students, or for personnel on the ground (unmounted) is unsupported by the evidence of injury types presented in the current study and others [13,19,20]. However, the two incidents involving horse riding as part of animal husbandry activities, indicates that their proactive use in this situation is prudent. It is the authors’ view that in addition to relying upon protective equipment (where evidence for its use exists), the emphasis placed on educating university students about occupational safety requirements, and specific preventative measures relevant to horse handling and interaction should be maintained [10,13,18]. 

Overall, these differences between the mechanism, bodily location, and nature of injuries between the populations highlight the markedly different characteristics of injury risk and type for university students, and the need for focused study of the risks to this group [2,17]. Recommendations regarding injury prevention therefore need to be specifically focused on addressing the risks faced by student in university programs. Ideally these should encompass considerations of the specific interactions of students with horses, rather than generally applying those of populations engaged in the equine industry professionally or recreationally [15,16,18]. Not only does this refer to technical risk mitigation, but also socio-cultural mitigation in relation to less tangible risks such as student-specific authority gradients that may discourage students following safety protocol during extramural placements [13,17].

The sample response rate was considered representative of the study population. However caution should be exercised in the broader application of these findings. The survey was retrospective and self-reported, comprising stated attitudes and behaviors within an Australian cultural context, and relied on the ability of the students to recall the details of incidents. Prospective data collection using a standardized approach is recommended to evaluate incidents and near misses, as well as the socio-cultural aspects of accident and safety culture [13], and attitudes among students across programs, institutions and time.

## 5. Conclusions 

Students engaged in university programs involving equine husbandry and veterinary education are at risk of injury, particularly when specific cohorts are engaged in extramural activities. However, injuries are generally not severe in nature, and students report that they normally recovered fully. Based upon injury patterns reported, consideration should be given to the use of protective boots, gloves, and possibly padded coveralls for on-campus and extramural equine-related learning activities. From this report describing the types of HRI in students, it is evident that published data for the practicing veterinarians, equine industry, or equestrian populations should not be relied upon make assumptions about the incidence, nature, and risk of injury to this population. Further study of animal associated risks to students during their education is warranted, and should acknowledge and address the social as well as technical dimensions of safety and risk management [13].
